# Supplementary material for: Efficient Synthesis of PVDF/PI Side-by-Side Bicomponent Nanofiber Membrane with Enhanced Mechanical Strength and Good Thermal Stability
Source: Nanomaterials (Basel). 2018 Dec 29;9(1):39. doi: 10.3390/nano9010039 (PMC6359095; doi:10.3390/nano9010039)
Supplement: Supplementary file 1 [file nanomaterials-09-00039-s001.pdf]

*Supplementary Materials*

# Efficient Synthesis of PVDF/PI Side-by-Side Bicomponent Nanofiber Membrane with Enhanced Mechanical Strength and Good Thermal Stability

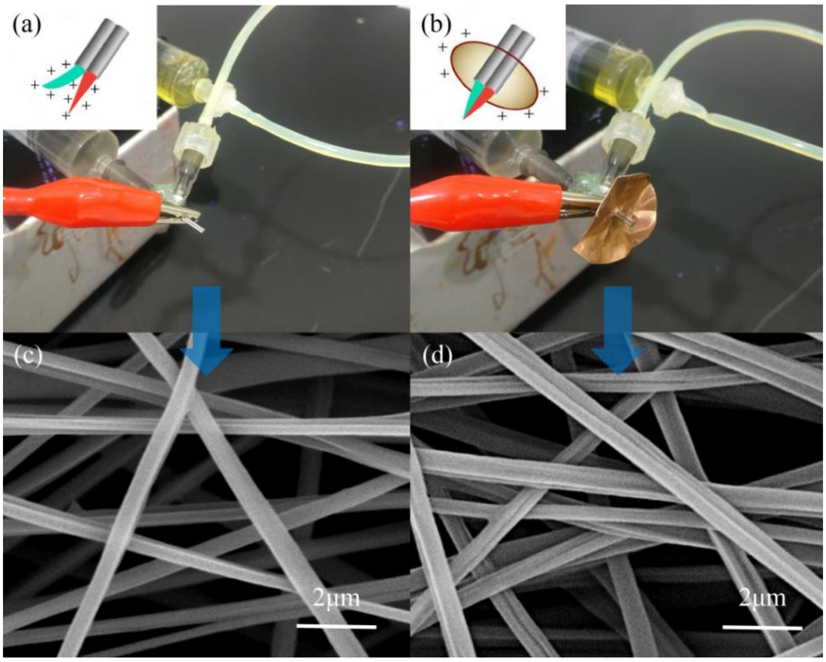


**Figure S1.** The schematic diagram of the electrospinning device before (**a**) and after (**b**) improvement; The corresponding SEM images of PVDF/PI side-by-side fibers before (**c**) and after (**d**) improvement.

In order to identify the two components in PVDF/PI side-by-side fibers, fluorescent labeling experiment was introduced. Fluorescein (0.5 wt %) was added to the PVDF solution, which shows intense green fluorescence. And Rhodamine B (0.5 wt %) was put into the PI solution that possess strong red fluorescence. In Figure S2, green PVDF and orange PI can be evidently observed under fluorescence microscope.

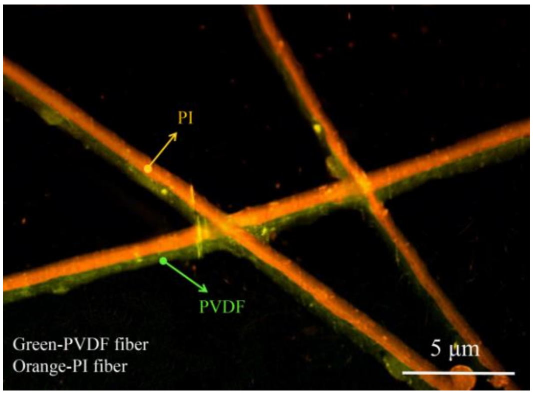


**Figure S2.** Fluorescence microscopy image: green-PVDF fiber, orange-PI fiber.
